# Supplementary material for: Rhabdomyolysis related to acute recreational drug toxicity—A Euro-DEN study
Source: PLoS One. 2021 Mar 11;16(3):e0246297. doi: 10.1371/journal.pone.0246297 (PMC7951866; doi:10.1371/journal.pone.0246297)

PLOS ONE

Rhabdomyolysis related to acute recreational drug toxicity – a Euro-DEN study.

--Manuscript Draft--

**Manuscript Number:**	PONE-D-20-18319
**Article Type:**	Research Article
**Full Title:**	Rhabdomyolysis related to acute recreational drug toxicity – a Euro-DEN study.
**Short Title:**	Rhabdomyolysis following acute recreational drug toxicity
**Corresponding Author:**	Piotr Maciej Kabata, M.D. Ph.D.<br>Pomorskie Centrum Toksykologii<br>Gdańsk, POLAND
**Keywords:**	Rhabdomyolysis; Recreational drugs; Acute Harm; Clinical toxicology; outcome.
**Abstract:**	Backgr o und : This study was conducted to retrospectively assess the relationships between: rhabdomyolysis (quantified by creatine kinase (CK) activity) and kidney injury (quantified by serum creatinine concentration), sex, age, body temperature on admission, presence of seizures, and agitation or aggression in patients presenting to the Emergency Department with acute recreational drug toxicity. We also investigated the association with the substances ingested.<br><br>Methods: All presentations to the 16 sentinel Euro-DEN centres in 10 European countries with acute recreational drug toxicity during the first year of the Euro-DEN study (October 2013 to September 2014) were considered. Cases that had abnormal CK activity recorded as part of routine clinical care were divided into 3 cohorts depending on peak CK activity. Cases with normal CK activity were included as a co ntrol group (4 th cohort).<br><br>Results: Only 1,015 (18.4%) of the 5,529 Euro-DEN presentations had CK activity concentration recorded. Of this group 353 (34.8%) had also creatinine concentration measured. There were 375 (36.9%) with minor rhabdomyolysis, 69 (6.8%) with moderate rhabdomyolysis, and 24 (2.4%) with severe rhabdomyolysis; 547 (53.9%) were included in the control group. There was a positive correlation between CK activity and creatinine concentration (correlation coefficient r=0.7 1 , p<0.0001). There was no correlation between CK activity and body temperature at the time of presentation to the ED (correlation coefficient r=0.07, p = 0.0 3). There was a positive correlation between CK activity and length of stay in the hospital (r=0.31, p<0.001). There was no association between CK activity and the presence of seizures (p=0.33) or agitation/aggression (p=0.45), patients age (p=0.4) or sex (p=0.25). The 5 most common agents amongst patients presenting with rhabdomyolysis were: cocaine (n=107; 22.9% presentations), amphetamine (76; 16.2%), cannabis (74; 15.8%), GHB/GBL (72; 15.4%) and heroin (67; 14.3%). In the group without rhabdomyolysis the most common agents were: cocaine (n=122 presentations; 223%), cannabis (117; 21.4%), GHB/GBL (81; 14.8%), heroin (70; 12.1%) and amphetamine (66; 11.7%).<br><br>Conclusions : Abnormal values of CK activity occurred in almost half (46.1%) of presentations to the Emergency Department with acute recreational drug toxicity in whom CK activity was measured; however, severe rhabdomyolysis is seen in only a small minority (2.4%). Those with rhabdomyolysis are at significantly higher risk of kidney injury and have a longer length of hospital stay.
**Order of Authors:**	Wojciech Waldman
	Piotr Maciej Kabata, M.D. Ph.D.
	Alison M Dines
	David M Wood
	Christopher Yates
	Fridtjof Heyerdahl
	Knut Erik Hovda

Powered by Editorial Manager® and ProduXion Manager® from Aries Systems Corporation

	Isabelle Giraudon
	Paul I Dargan
	Jacek Sein Anand
**Additional Information:**	
**Question**	**Response**
**Financial Disclosure**<br><br>Enter a financial disclosure statement that describes the sources of funding for the work included in this submission. Review the submission guidelines for detailed requirements. View published research articles from *PLOS ONE* for specific examples.<br><br>This statement is required for submission and **will appear in the published article** if the submission is accepted. Please make sure it is accurate.<br><br>**Unfunded studies**<br>Enter: *The author(s) received no specific funding for this work.*<br><br>**Funded studies**<br>Enter a statement with the following details:<br>• Initials of the authors who received each award<br>• Grant numbers awarded to each author<br>• The full name of each funder<br>• URL of each funder website<br>• Did the sponsors or funders play any role in the study design, data collection and analysis, decision to publish, or preparation of the manuscript?<br>• **NO** - Include this sentence at the end of your statement: *The funders had no role in study design, data collection and analysis, decision to publish, or preparation of the manuscript.*<br>• **YES** - Specify the role(s) played.<br><br>* typeset	With financial support from the DPIP/ISEC Programme of the European Union and the European Monitoring Centre for Drugs and Drug Addiction.<br>All the authors had funding from the European Commission through the Euro-DEN project except ML and EL, whose costs were co-funded by the Swiss Centre for Applied Human Toxicology (SCAHT) and KP, IG and RS.
**Competing Interests**<br><br>Use the instructions below to enter a competing interest statement for this submission. On behalf of all authors, disclose any competing interests that could be perceived to bias this	The authors have declared that no competing interests exist.

work—acknowledging all financial support and any other relevant financial or non-financial competing interests.<br><br>This statement **will appear in the published article** if the submission is accepted. Please make sure it is accurate. View published research articles from *PLOS ONE* for specific examples.<br><br>**NO authors have competing interests**<br><br>Enter: *The authors have declared that no competing interests exist.*<br><br>**Authors with competing interests**<br><br>Enter competing interest details beginning with this statement:<br><br>*I have read the journal's policy and the authors of this manuscript have the following competing interests: [insert competing interests here]*<br><br>* typeset	
**Ethics Statement**<br><br>Enter an ethics statement for this submission. This statement is required if the study involved:<br><br>• Human participants<br>• Human specimens or tissue<br>• Vertebrate animals or cephalopods<br>• Vertebrate embryos or tissues<br>• Field research<br><br>Write "N/A" if the submission does not require an ethics statement.<br><br>General guidance is provided below. Consult the submission guidelines for detailed instructions. **Make sure that all information entered here is included in the Methods section of the manuscript.**	N/A

**Format for specific study types**<br><br>**Human Subject Research (involving human participants and/or tissue)**<br>• Give the name of the institutional review board or ethics committee that approved the study<br>• Include the approval number and/or a statement indicating approval of this research<br>• Indicate the form of consent obtained (written/oral) or the reason that consent was not obtained (e.g. the data were analyzed anonymously)<br><br>**Animal Research (involving vertebrate animals, embryos or tissues)**<br>• Provide the name of the Institutional Animal Care and Use Committee (IACUC) or other relevant ethics board that reviewed the study protocol, and indicate whether they approved this research or granted a formal waiver of ethical approval<br>• Include an approval number if one was obtained<br>• If the study involved *non-human primates*, add *additional details* about animal welfare and steps taken to ameliorate suffering<br>• If anesthesia, euthanasia, or any kind of animal sacrifice is part of the study, include briefly which substances and/or methods were applied<br><br>**Field Research**<br><br>Include the following details if this study involves the collection of plant, animal, or other materials from a natural setting:<br>• Field permit number<br>• Name of the institution or relevant body that granted permission	
**Data Availability**<br><br>Authors are required to make all data underlying the findings described fully available, without restriction, and from the time of publication. PLOS allows rare exceptions to address legal and ethical concerns. See the PLOS Data Policy and FAQ for detailed information.	No - some restrictions will apply

Powered by Editorial Manager® and ProduXion Manager® from Aries Systems Corporation

A Data Availability Statement describing where the data can be found is required at submission. Your answers to this question constitute the Data Availability Statement and **will be published in the article**, if accepted.<br><br>**Important:** Stating 'data available on request from the author' is not sufficient. If your data are only available upon request, select 'No' for the first question and explain your exceptional situation in the text box.<br><br>Do the authors confirm that all data underlying the findings described in their manuscript are fully available without restriction?	
**Describe where the data may be found in full sentences. If you are copying our sample text, replace any instances of XXX with the appropriate details.**<br><br>• If the data are **held or will be held in a public repository**, include URLs, accession numbers or DOIs. If this information will only be available after acceptance, indicate this by ticking the box below. For example: *All XXX files are available from the XXX database (accession number(s) XXX, XXX.).*<br>• If the data are all contained **within the manuscript and/or Supporting Information files**, enter the following: *All relevant data are within the manuscript and its Supporting Information files.*<br>• If neither of these applies but you are able to provide **details of access elsewhere**, with or without limitations, please do so. For example:<br><br>*Data cannot be shared publicly because of [XXX]. Data are available from the XXX Institutional Data Access / Ethics Committee (contact via XXX) for researchers who meet the criteria for access to confidential data.*<br><br>*The data underlying the results presented in the study are available from (include the name of the third party*	The data may be obtained from the authors, after acceptance of EuroDEN research group Steering Committee. Tha data have been obtained from a number of individual centres, and collated by the Steering Committee.

and contact information or URL).<br>• This text is appropriate if the data are owned by a third party and authors do not have permission to share the data.<br><br>* typeset	
Additional data availability information:	

Powered by Editorial Manager® and ProduXion Manager® from Aries Systems Corporation

Manuscript
Click here to access/download;Manuscript;EuroDEN_rhabdomyolysis_paper

Rhabdomyolysis related to acute recreational drug toxicity – a Euro-DEN study.

Wojciech Waldman (1,2), Piotr M Kabata (2), Alison M Dines (3), David M Wood (3,4), Christopher Yates (5), Fridtjof Heyerdahl (6), Knut Erik Hovda (6), Isabelle Giraudon (7), Euro-DEN Research Group (8), Paul I Dargan (3,4), Jacek Sein Anand (1,2).

(1) Department of Clinical Toxicology, Medical University of Gdansk, Gdansk, Poland
(2) Pomeranian Centre of Toxicology, Gdansk, Poland (3) Clinical Toxicology, Guy's and St Thomas' NHS Foundation Trust and King's Health Partners, London, UK
(4) Faculty of Life Sciencesand Medicine, King's College London, London, UK
(5) Hospital Universitari Son Espases, Palma de Mallorca, Spain
(6) Medical Division, Oslo University Hospital, Oslo, Norway
(7) European
Monitoring Centre for Drugs and Drug Addiction (EMCDDA), Lisbon, Portugal
(8) Euro-DEN Research Group.

Acknowledgments:
Euro-DEN Research Group Members:
Lucie Chevillard, Florian Eyer, Miguel Galicia, Catalina Homar, Gesche Jürgens, Evangelia Liakoni, ME Liechti, Bruno Mégarbane, Oscar Miro, Adrian Moughty, Niall O'Connor, Raido Paasma, Kristiina Põld, Odd Martin Vallersnes, WS Waring

Keywords: Rhabdomyolysis; Recreational drugs; Acute Harm; Clinical toxicology; Outcome.

Abbreviations: AKI: acute kidney injury; ALT: alanine aminotransferase; AST: aspartate aminotransferase; CK: creatine kinase; ED: emergency department; Euro-DEN: European Drug Emergencies Network; GBL: γ-butyrolactone; GHB: γ-hydroxybutyric acid; IQR: interquartile range; KDIGO: Kidney Disease: Improving Global Outcomes; LOS: Length of stay in hospital; PSS: Poisoning Severity Score.

Abstract:

Background: This study was conducted to retrospectively assess the relationships between: rhabdomyolysis (quantified by creatine kinase (CK) activity) and kidney injury (quantified by serum creatinine concentration), sex, age, body temperature on admission, presence of seizures, and agitation or aggression in patients presenting to the Emergency Department with acute recreational drug toxicity. We also investigated the association with the substances ingested.

Methods: All presentations to the 16 sentinel Euro-DEN centres in 10 European countries with acute recreational drug toxicity during the first year of the Euro-DEN study (October 2013 to September 2014) were considered. Cases that had abnormal CK activity recorded as part of routine clinical care were divided into 3 cohorts depending on peak CK activity. Cases with normal CK activity were included as a control group (4th cohort).

Results: Only 1,015 (18.4%) of the 5,529 Euro-DEN presentations had CK activity concentration recorded. Of this group 353 (34.8%) had also creatinine concentration measured. There were 375 (36.9%) with minor rhabdomyolysis, 69 (6.8%) with moderate rhabdomyolysis, and 24 (2.4%) with severe rhabdomyolysis; 547 (53.9%) were included in the control group. There was a positive correlation between CK activity and creatinine concentration (correlation coefficient $r=0.71$, $p<0.0001$). There was no correlation between CK activity and body temperature at the time of presentation to the ED (correlation coefficient $r=0.07$, $p=0.03$). There was a positive correlation between CK activity and length of stay in the hospital ($r=0.31$, $p<0.001$). There was no association between CK activity and the presence of seizures ($p=0.33$) or agitation/aggression ($p=0.45$), patients age ($p=0.4$) or sex ($p=0.25$). The 5 most common agents amongst patients presenting with rhabdomyolysis were: cocaine (n=107; 22.9% presentations), amphetamine (76; 16.2%), cannabis (74; 15.8%), GHB/GBL (72; 15.4%) and heroin (67; 14.3%). In the group without rhabdomyolysis the most common agents were: cocaine (n=122 presentations; 223%), cannabis (117; 21.4%), GHB/GBL (81; 14.8%), heroin (70; 12.1%) and amphetamine (66; 11.7%).

It would be more interesting to show distribution of rhabdomyolysis acording to agents abused

Conclusions: Abnormal values of CK activity occurred in almost half (46.1%) of presentations to the Emergency Department with acute recreational drug toxicity in whom CK activity was measured; however, severe rhabdomyolysis is seen in only a small minority (2.4%). Those with rhabdomyolysis are at significantly higher risk of kidney injury and have a longer length of hospital stay.

Declarations

Ethics approval and consent to participate

The study involved retrospective analysis of anonymised clinical data records. The need for approval was waived by the ethical committees of respective centres participating in the study.

Availability of data and materials

The data that support the findings of this study are available from Euro-DEN research group but restrictions apply to the availability of these data, which were used under license for the current study, and so are not publicly available. Data are however available from the authors upon reasonable request and with permission of Euro-DEN research group steering committee.

Competing interests

The authors declare that they have no competing interests.

Funding:
With financial support from the DPIP/ISEC Programme of the European Union and the European Monitoring Centre for Drugs and Drug Addiction.
All the authors had funding from the European Commission through the Euro-DEN project except ML and EL, whose costs were co-funded by the Swiss Centre for Applied Human Toxicology (SCAHT) and KP, IG and RS.

Introduction

Rhabdomyolysis is a syndrome that is characterized by the disintegration of striated muscle and the leakage of muscle-cell contents including myoglobin and creatine kinase (CK). The first detailed reports of rhabdomyolysis, related to the crush syndrome, and complicated with acute renal failure, were presented by Bywaters and Beall in the 1940s [1,2]. CK is more reliable than myoglobin in assessing the presence and intensity of damage to the muscles because overall CK degradation and removal are slow, the concentration of CK remains elevated much longer and in a more consistent manner than that of myoglobin. Definitions of rhabdomyolysis vary from that requiring CK elevation greater than approximately five times the upper limit of normal to more recent definitions requiring CK elevation greater than 50 times the upper limit of normal with renal insufficiency [3]. Rhabdomyolysis is a serious condition, which may lead to life threatening complications.

The causes of rhabdomyolysis can be divided into hereditary and acquired ones. The acquired causes are classified as traumatic and non-traumatic. Non-traumatic causes are the most common during peacetime and include psychoactive drugs, alcohol abuse, and many others. The medical literature is filled with case reports of rhabdomyolysis resulting from snake and spider bites [4–6] to drugs like cocaine, methamphetamine [7–11], 3,4-methylenedioxymethamphetamine (MDMA) [12,13] and plant toxins [14].

Despite the fact that at least 150 medications and toxins have been described which may lead to rhabdomyolysis, recreational drugs and alcohol are the most common causes [15]. Many factors are known to contribute to development of rhabdomyolysis including seizures, agitation or aggression, immobilisation, excessive muscle activity, hypo- or hyperthermia [3,15–20], and potentially also direct substance-associated toxic effects in susceptible persons [21]. There have been no large, multicentre studies on the incidence of rhabdomyolysis in acute recreational drug toxicity.

The European Drug Emergencies Network (Euro-DEN) project collects data on presentations to sentinel Emergency Departments (ED) in Europe in whom the primary reason for presentation is acute recreational drug toxicity [22,23]. During the first year from October 2013 to September 2014 data was collected from 16 sentinel centres in 10 European countries. The aim of this study was to use data from the Euro-DEN project to determine the relationship between maximum CK activity and factors frequently associated with rhabdomyolysis (creatinine concentration as a marker of acute kidney injury (AKI), length of stay in hospital, patients' temperature at admission, presence of seizures, agitation or aggression, hyperthermia) as well as others that might influence it (substances used by the patient and their number in case of multisubstance abuse).

Materials and methods:

Data collection:

All acute recreational drug toxicity presentations to the 16 sentinel ED in 10 European countries participating in the first year (October 2013 to September 2014) of the Euro-DEN project were included retrospectively using standard Euro-DEN methodology [22]. Patients presenting with other main complaints (including abstinence syndromes) who were under influence of psychoactive substances were excluded. Presentations involving intoxication with ethanol without other

Any justification for restricting the retrospective study to Oct 2013 to Sept 2014? Are there more recent data?

coingested psychoactive substances was excluded from the study. In case of lack of information regarding used substance from patient history or laboratory results, collective label “unknown substance” was used.
After completing first year of data collection, gathered dataset was revised and some of the variables (including creatinine concentration and CK activity) were removed.
In the Euro-DEN dataset only highest recorded CK activity and serum creatinine concentrations measured during patients stay were recorded [22,23].

Data analysis:
The dataset was converted from the Euro-DEN Excel spreadsheet to a comma separated values file. Data wrangling, analysis and visualisations for this analysis were performed in R programming language for statistics [24] with use of modules: ggplot2 [25], dplyr [26], gridExtra [27], reshape2 [28]. Analysis was performed by the Euro-DEN centre located in Gdansk Poland.

Only 1015 (18.4%) of the 5,529 Euro-DEN presentations over the study period had CK activity recorded in the database and included in the analysis. Parameters analysed were: time from use to presentation, self-reported number of agents used, what agents were used (reported), presence of seizures, body temperature on admission, presence of agitation/aggression, maximum CK activity recorded, maximum creatinine recorded, length of stay in hospital (LOS). All the missing values were filled with ‘NA’ value.

For further analysis presentations were divided into 4 cohorts depending on maximum CK activity. The values used for the partitioning of the data were based on values used in Poison Severity Score (PSS) [29]: 1. no rhabdomyolysis (CK below 250 IU/L), 2. minor rhabdomyolysis (CK 251 – 1,500 IU/L), 3. moderate rhabdomyolysis (CK 1,501 – 10,000 IU/L), 4. severe rhabdomyolysis (CK above 10,000 IU/L). It should be noted, that the severity of rhabdomyolysis does not have to be consistent with severity of poisoning, which was not assessed in this analysis.
Fragmented groups of substances sharing chemical and toxicological properties (for example benzodiazepines) were treated as one causative agent during the analysis.

The use of range and interquartile range as measure of data spread suggest that the data were not normally distributed. Hence the use of parametric test such as pearson's moment correlation and ANOVA for analysis may not be appropriated.

Statistical analysis:
Threshold for statistical significance for the testing was established at $p < 0.01$ (probability of type I error smaller than 1%). We used both range and interquartile range (IQR) as measures of data spread. Correlations were tested using Pearson's product-moment correlation coefficient. For assessing relationship between multiple factors, we used analysis of variance (ANOVA). Where analysis of means between the groups was needed, Welch Two Sample t-test was performed.

Results:
Only presentations with CK activity recorded (N = 1015) were included in the analysis. The median (IQR; range) CK was 228 (124.5 – 448.0; 30 – 169,700) IU/L.

CK (IU/L)	**≤ 250 IU/l**	**250 - 1500 IU/l**	**1501 – 10000 IU/l**	**CK >10000 IU/l**	**Total**
**n**	547 (53.9%)	375 (36.9%)	69 (6.8%)	24 (2.4%)	1015 (100%)
**Min**	30	252	1522	10036	30

**Max**	250	1397	9441	169700	169700
**Median**	132.0	441.0	2595.0	21934.0	228.0
**IQR**	94.0 – 183.0	319.0 – 684.5	1986.0 – 4030.0	16760.0 – 63640.0	124.0 – 488.0

Table 1. Creatine Kinase (CK) results in the 1015 Euro-DEN Presentations

As shown in Table 1, 46.1% of this cohort had a CK > 250 IU/L, the majority of whom had minor rhabdomyolysis.

CK vs.Creatinine:

Of the 1015 patients included in our analysis only 353 (39,11%) had both CK and creatinine concentration recorded.

In the total studied population, median creatinine was 0.90 mg/dl (min 0.40 mg/dl, max 6.94 mg/dl, IQR 0.72 – 1.10 mg/dl). For patients with no rhabdomyolysis median creatinine was 0.80 mg/dl (min 0.41 mg/dl, max 1.47 mg/dl, IQR 0.69 – 0.91 mg/dl). For patients with rhabdomyolysis median creatinine was 0.99 mg/dl (min 0.40 mg/dl, max 6.94 mg/dl, IQR 0.81 – 1.20 mg/dl).

Median creatinine of patients with minor rhabdomyolysis was 0.91 mg/dl (min 0.40 mg/dl, max 1.75 mg/dl, IQR 0.80 – 1.10 mg/dl). Median creatinine of patients with moderate rhabdomyolysis was 1.01 mg/dl (min 0.56 mg/dl, max 2.40 mg/dl, IQR 0.86 – 1.37 mg/dl). Median creatinine of patients with severe rhabdomyolysis was 1.65 mg/dl (min 0.54 mg/dl, max 6.94 mg/dl, IQR 0.95 – 2.97 mg/dl). The correlation between CK activity and creatinine was 0.71, p < 0,0001. In the patients with both creatinine and CK measured 44 (12.5%) had creatinine concentration of 1.2 mg/dl or higher, which was considered a marker of kidney injury.

The prevalence of kidney injury is presented in Table 2. The presence of rhabdomyolysis increased the odds of kidney injury 3.97 times.

What is your definition of Kidney injury ? This should be elaborated in the method

Kidney injury	**[ALL]** *N=353*	**No** *N=309*	**Yes** *N=44*	**Odds Ratio**	**p for odds ratio**	**p overall**
Cohort:						
NONE	188 (53.3%)	177 (57.3%)	11 (25.0%)	Reference	Reference	
MINOR	124 (35.1%)	109 (35.3%)	15 (34.1%)	2.20 [0.97;5.13]	0.058	<0.001
MODERATE	31 (8.78%)	19 (6.15%)	12 (27.3%)	9.95 [3.84;26.4]	<0.001	
SEVERE	10 (2.83%)	4 (1.29%)	6 (13.6%)	22.9 [5.57;106]	<0.001	
Rhabdomyolysis:						<0.001
No	188 (53.3%)	177 (57.3%)	11 (25.0%)	Reference	Reference	

This Table should be properly labelled. Also, distinguish between the exposure and outcome variable.

Yes	165 (46.7%)	132 (42.7%)	33 (75.0%)	3.97 [1.99;8.55]	<0.001	

The relationships between peak CK activity and peak creatinine concentration are shown in figure 1; as shown, there was a significant positive correlation between peak CK activity and creatinine concentration.

Figure 1. Correlation between CK activity and max serum creatinine concentrations. r - Pearson correlation coefficient, p - confidence level.

CK vs age: The interval is to wide to report mean. Median will be more appropriate

The youngest patient in the study was 12 years old, the oldest was 88 years old. Mean age of a patient was 30,7 years.

There was no significant correlation between the age of a patient and CK activity (p=0.41). report r

CK vs sex of a patient:

was

The study included 241 women and 774 men. Rhabdomyolysis has been observed in 68 (28,2%) women and 400 (51,7%) of male patients. The differences in CK activity were significant only for patients with no rhabdomyolysis. Include the test statistics

CK vs length of stay in hospital:

The length of stay in hospital (LOS) was longer in those with more severe rhabdomyolysis. In the total studied population, median LOS was 12.75 h (min 0.58 h, max 664 h, IQR 5.00 – 37.58 h). For patients with no rhabdomyolysis median LOS was 10.23 h (min 0.62 h, max 501 h, IQR 3.97 – 27.37 h). Median LOS of patients with minor rhabdomyolysis was 9.92 h (min 0.98 h, max 453.78 h, IQR 4.20 – 25.87 h). Median LOS of patients with moderate rhabdomyolysis was 25.25 h (min 0.58 h, max 664.00 h, IQR 12.63 – 55.91 h). Median LOS of patients with severe rhabdomyolysis was 108.98 h (min 2.81 h, max 661.15 h, IQR 43.09 – 255.80 h). The correlation between CK activity and LOS was 0.31, p<0,001.

CK vs. body temperature on admission:

Of 1015 patients included in our analysis body temperature on admission was measured in 933 (91.92%). The data indicates that there is no correlation between body temperature on admission and rhabdomyolysis (r = 0.07, p = 0.03).

CK vs. presence of seizures and agitation or aggression:

There was a no significant relationship between seizures being present at any time during the presentation and the severity of the rhabdomyolysis (Table 3).

	Total		No rhabdomyolysis		Mild rhabdomyolysis		Moderate rhabdomyolysis		Severe rhabdomyolysis	
Seizures	Yes	No	Yes	No	Yes	No	Yes	No	Yes	No
n (% of patients in cohort)	72 (7.1%)	941 (92.7%)	34 (6.2%)	512 (93.6%)	28 (7.5%)	347 (92.5%)	5 (7.3%)	64 (92.8%)	5 (20.8%)	18 (75.0%)

Median CK	266	225	135	131	483	440	2552	2624	22080	19450
Agitation / Aggression	Yes	No	Yes	No	Yes	No	Yes	No	Yes	No
n (% of patients in cohort)	390 (38.4%)	624 (61.5%)	176 (32.2%)	370 (67.6%)	165 (44.0%)	210 (56.0%)	37 (53.6%)	32 (46.4%)	12 (50.0%)	12 (50.0%)
Median	286	208	144	125	469	430	2365	2834	21380	22440

Table 3. CK activity, seizures at any time during the admission and agitation/aggression on admission. For 2 patients (1 in No rhabdomyolysis and 1 in Severe rhabdomyolysis cohort) there was no information regarding presence of seizures. For 1 patient (in No rhabdomyolysis cohort) there was no information regarding presence of agitation/aggression.

CK vs. drugs used:

As shown in Table 4 the drugs used by those with rhabdomyolysis were similar to those without rhabdomyolysis. It is difficult to interprete this table. It should be presented in a clearer format.

**Total population**		**Patients without rhabdomyolysis**		**Patients with rhabdomyolysis**		**Odds ratio**	**Odds ratio Confidence interval**
**Substance used**	**Frequency (N, %)**	**Substance used**	**Frequency (N, %)**	**Substance used**	**Frequency (N, %)**		
Cocaine	229 (22.6%)	Cocaine	122 (22.3%)	Cocaine	107 (22.9%)	1.03	0.79-1.34
Cannabis	192 (18.9%)	Cannabis	117 (21.4%)	Amphetamine	76 (16.2%)	1.34	0.97-1.89
GHB/GBL	153 (15.1%)	GHB/GBL	81 (14.8%)	Cannabis	74 (15.8%)	0.74	0.54-0.99
Amphetamine	142 (14.0%)	Heroin	70 (12.1%)	GHB/GBL	72 (15.4%)	1.04	0.75-1.44
Heroin	137 (13.5%)	Amphetamine	66 (11.7%)	Heroin	67 (14.3%)	1.12	0.8-1.58
Unknown substance	45 (4.3%)	Unknown substance	21 (3.8%)	Unknown substance	24 (5.1%)		

Table 4. 5 Most commonly reported psychoactive substances (as stated in medical records). For a reference number of unidentified agents was added.

There was no significant correlation between those values of CK activity in studied cohorts, and the number of agents (self-reported or tested) taken by the patient.

We have calculated the odds ratios for most frequently used substances. The calculation show, that the highest potential for rhabdomyolysis development is seen in amphetamine (OR 1.41).

Discussion:

Our results have shown that in almost half of the patients presenting to the ED with acute recreational drug toxicity in whom clinicians decided to measure CK activity due to higher risk of rhabdomyolysis, had elevated CK activities. This result might be biased by the fact that probably some of the centres measured CK activity only in patients that either clinically showed signs of muscle injury or their history suggested possible development of the condition. The majority of these had minor or moderate rhabdomyolysis, and only 2.4% developed severe rhabdomyolysis. There was a positive association between creatinine concentrations and the severity of rhabdomyolysis. However, the median creatinine concentration was elevated only in patients with CK activities >10 000 U/L. Due to study design, only highest creatinine concentration was recorded, thus it was not possible to check the patients against Kidney Disease: Improving Global Outcomes (KDIGO) acute kidney injury criteria. Data were also not available on the need for renal replacement therapy.

The mechanism of rhabdomyolysis in acute recreational drug toxicity is variable - some drugs have direct myocytotoxic effects, whilst others can increase physical activity to deleterious levels, produce ischemia due to arterial vasoconstriction and precipitate seizures or hyperthermia [21,30-35]. Depressant intoxication can also lead to immobilisation and rhabdomyolysis related to compression and ischemic injury. For example, in a small previous study CK levels were correlated with the duration of coma and coma was more prolonged in patients with combined use of γ-hydroxybutyric acid (GHB) and stimulants [33,34].

Although illicit drugs are well-described precipitants of rhabdomyolysis [36,37], there is limited data on the frequency of rhabdomyolysis in patients presenting with acute recreational drug toxicity. Due to different study methodology, inclusion criteria based upon emergency or hospital physician coding, and variable definitions of “renal failure”, these studies may be difficult to compare. Importantly none of studies included patients discharged from ED or performed follow-up of patients to identify adverse events post-hospital discharge. We have found only one study, presented by Grunau et al., that compared CK activities of patients who were admitted to the hospital to those measured in all ED presentations. In this study, 400 cases of rhabdomyolysis, defined as CK level greater than 1 000 U/l, were found by the authors in 9,509 patients who had CK activity test ordered out of 235,947 ED visits. 30-day follow-up was performed after ED treatment. 35% of patients were discharged home from ED. The most common ED discharge diagnoses were related to recreational drug use, infections, and traumatic or musculoskeletal complains. Within 30 days 21 (5.3%) patients died with 18 (4.5%) requiring hemodialysis. AKI occurred in 151 (38%) patients. In this work, the authors found that higher CK values were not associated with worse outcomes and concluded that initial creatinine was the best predictor of outcome [39]. Completely different results were obtained by Janković et al. who analysed the incidence of rhabdomyolysis in acute poisoning with different agents in Serbia. Rhabdomyolysis occurred at a relatively high rate in 125 out of 656 patients during a one-year period (19%). Rhabdomyolysis was mainly mild (61%) (CK from 250 to 1500 U/l), or moderate (36%) (CK from 1500 to 10000), and only in 3% of the patients was rhabdomyolysis severe. The incidence of rhabdomyolysis was highest in poisonings involving opiates (41%), pesticides (38%), neuroleptics (26%), anticonvulsants (26%), ethanol (20%) and gases (19%). Psychotropic agents were the most common causes of poisonings, and consequently of rhabdomyolysis. One limitation of this study was that rhabdomyolysis was diagnosed based only on biochemical indicator (CK), without clinical signs and symptoms [41].

As there are no universal CK activity criteria to establish diagnosis of rhabdomyolysis, we used the Poisoning Severity Score to determine severity of rhabdomyloysis. Partitioning to cohorts was important for the analysis, due to data spread (maximum CK activity was 5657 times higher than the minimum). Cohorts with normal CK activity vs. elevated were similar in size (N=547 vs. N=468). A group with elevated CK represented 8.5% of total and 46.2% of patients where CK was measured. Only 353 patients had both CK and creatinine measured.

Literature data show that higher CK activities correlated with degree of muscle injury, but correlated only slightly with the development of AKI or mortality [15,36,40,42–46]. The incidence of AKI secondary to rhabdomyolysis varies from 10% to 59%, whilst it is estimated that 5% to 15% of AKI cases can be attributed to rhabdomyolysis [15,16,36,38,40,42-44]. In previous studies on

cocaine-intoxicated patients, the prevalence of AKI as the consequence of rhabdomyolysis was reported as 24% to 33% [9,11]. Based on our analysis we have observed a statistically significant positive correlation between highest CK activity and highest creatinine concentration in a general population as well as in most of the cohorts. The severity of rhabdomyolysis was associated with longer hospitalisation and thus could lead to higher treatment costs.

Our study did not show any correlation between the number of substances involved and rhabdomyolysis. The most common agents used by patients who developed rhabdomyolysis, were the same as those in patients without rhabdomyolysis with the 5 most common being cocaine, cannabis, GHB/GBL (γ-butyrolactone) amphetamine and heroine. Cocaine is a known factor contributing to development of muscular injury [9,11,38]. A PubMed search for "cannabis+rhabdomyolysis" showed only 8 results, of which only one was a report of connection between cannabis use and incidence of rhabdomyolysis, however the direct cause of the injury was falling asleep while sitting cross-legged. In our data, only 12 patients having seizures have used cannabis. Of this group 9 had no rhabdomyolysis and 3 minor rhabdomyolysis, which supports that observation. There is only a limited data on relationship between GHB/GBL and rhabdomyolysis in the literature, and most were reported cases involving muscle injury during withdrawal syndrome [33, 34, 47]. Considering the clinical effect of GHB/GBL, it can be assumed that rhabdomyolysis is caused by patient's immobility for prolonged periods. It should be noted, that MDMA was the most prevalent substance found in patients who developed severe rhabdomyolysis, however this group was relatively small, which might have influenced the result.

Limitations of the study:

The study was designed as a retrospective analysis of a large volume of clinical history records. Laboratory test data (CK activity and creatinine) were only recorded if these were taken as part of routine clinical care; this could potentially lead to a selection bias in the studied data as the patients with clinical signs of rhabdomyolysis would be tested more frequently. No data regarding physical activity in days before presentation was gathered, opening possibility to including patients who developed exertional rhabdomyolysis in the group. Due to the study design only the highest recorded CK activity and creatinine concentration was included, and data were not collected on previous renal function to be able to confirm that any kidney injury present was AKI rather than underlying chronic kidney disease. As the data were obtained from hospitals information systems, it was not possible to assure consistency of CK activity and creatinine concentration analytical methods. Information on agents used was based largely on patient self-report with laboratory confirmation in a minority; it should be noted however, that work by Liakoni et al. has shown that patient self-report is reliable particularly for traditional illicit drugs [48]. The data entered into the data collection tool were taken from medical histories and it was difficult to differentiate reported drug use from analytically confirmed in post-hoc analysis.

Due to revision of gathered dataset variables, we do not have information on CK activities and creatinine concentrations from EuroDEN presentations after first 12 months of the study.

Conclusions:

Abnormal values of CK activity may occur in up to half of presentations to the Emergency Department following acute recreational drug toxicity whose clinical signs suggest muscle injury

but severe rhabdomyolysis is much less common. Patients with rhabdomyolysis are at significantly higher risk of kidney injury (3.97 times higher, and 22.9 times higer for severe rhabdomyolysis) and spend more time in the hospital with a length of stay that is directly proportional to CK activity. Therefore, we suggest that it is valuable for patient management to check serum CK activity and creatinine concentrations in patients presenting to the Emergency Department with severe acute recreational drug toxicity as a part of routine laboratory testing.

Literature:

1. Bywaters EG, Beall D. Crush Injuries with Impairment of Renal Function. Br Med J. BMJ Publishing Group; 1941;1: 427–32. Available: http://www.ncbi.nlm.nih.gov/pubmed/20783577
2. Bywaters EGL. Experimental crushing injury. Surg Gynecol Obs. 1942;75: 612–627.
3. Thompson PD, Clarkson P, Karas RH. Statin-associated myopathy. Jamae. American Medical Association; 2003;289: 1681–1690.

4. Bush SP, Jansen PW. Severe rattlesnake envenomation with anaphylaxis and rhabdomyolysis. Ann Emerg Med. Elsevier; 1995;25: 845–848.
5. Bush SP, Siedenburg E. Neurotoxicity associated with suspected southern Pacific rattlesnake (Crotalus viridis helleri) envenomation. Wilderness Environ Med. Elsevier; 1999;10: 247–249.
6. Cohen J, Bush S. Case report: compartment syndrome after a suspected black widow spider bite. Ann Emerg Med. Elsevier; 2005;45: 414–416.
7. Brody SL, Wrenn KD, Wilber MM, Slovis CM. Predicting the severity of cocaine-associated rhabdomyolysis. Ann Emerg Med. Elsevier; 1990;19: 1137–1143.
8. Richards JR, Johnson EB, Stark RW, Derlet RW. Methamphetamine abuse and rhabdomyolysis in the ED: a 5-year study. Am J Emerg Med. Elsevier; 1999;17: 681–685.
9. Roth D, Alarcón FJ, Fernandez JA, Preston RA, Bourgoignie JJ. Acute rhabdomyolysis associated with cocaine intoxication. N Engl J Med. 1988;319: 673–7. doi:10.1056/NEJM198809153191103
10. Singhal PC, Rubin RB, Peters A, Santiago A, Neugarten J. Rhabdomyolysis and acute renal failure associated with cocaine abuse. J Toxicol Clin Toxicol. Taylor & Francis; 1990;28: 321–330.
11. Welch RD, Todd K, Krause GS. Incidence of cocaine-associated rhabdomyolysis. Ann Emerg Med. 1991;20: 154–7. Available: http://www.ncbi.nlm.nih.gov/pubmed/1996798
12. Cunningham M. Ecstasy-induced rhabdomyolysis and its role in the development of acute renal failure. Intensive Crit care Nurs. Elsevier; 1997;13: 216–223.
13. Traub SJ, Hoffman RS, Nelson LS. The "ecstasy" hangover: hyponatremia due to 3, 4-methylenedioxymethamphetamine. J Urban Heal. Springer; 2002;79: 549–555.
14. Sein Anand J, Kabata P, Barwina M Waldman W. Some Aspects of Intoxication with Tricholoma equestre. Clin Toxicol. 2011;49: 230.

15. Melli G, Chaudhry V, Cornblath DR. Rhabdomyolysis: an evaluation of 475 hospitalized patients. Medicine (Baltimore). 2005;84: 377–85.
16. Larbi EB. Drug-induced rhabdomyolysis. Ann Saudi Med. 18: 525–30. Available: http://www.ncbi.nlm.nih.gov/pubmed/17344731
17. Allison RC, Bedsole DL. The other medical causes of rhabdomyolysis. Am J Med Sci. Elsevier; 2003;326: 79–88.
18. Warren J, Blumbergs P, Thompson P. Rhabdomyolysis: a review. Muscle Nerve. 2002;25: 334–47.
19. Prendergast BD, George CF. Drug-induced rhabdomyolysis - mechanisms and management. Postgrad Med J. 1993;69: 333–336.
20. Zager RA. Rhabdomyolysis and myohemoglobinuric acute renal failure. Kidney Int. Elsevier; 1996;49: 314–326.
21. Zhou, X., Luethi, D., Sanvee, G. M., Bouitbir, J., Liechti, M. E., & Krähenbühl, S. (2019). Molecular Toxicological Mechanisms of Synthetic Cathinones on C2C12 Myoblasts. International Journal of Molecular Sciences, 20(7), 1561.
22. Dines AM, Wood DM, Yates C, Heyerdahl F, Hovda KE, Giraudon I, et al. Acute recreational drug and new psychoactive substance toxicity in Europe: 12 months data collection from the European Drug Emergencies Network (Euro-DEN). Clin Toxicol. 2015;53: 893–900. doi:10.3109/15563650.2015.1088157
23. Final Report of the European Drug Emergencies Network (Euro - DEN) March 2015. 2015.
24. R Core Team. R: A Language and Environment for Statistical Computing [Internet]. Vienna, Austria: R Foundation for Statistical Computing; 2016. Available: https://www.r-project.org/
25. Wickham H. ggplot2: Elegant Graphics for Data Analysis [Internet]. New York: Springer-Verlag New York; 2009. Available: http://ggplot2.org
26. Wickham H, Francois R. dplyr: A Grammar of Data Manipulation [Internet]. 2016. Available: https://cran.r-project.org/package=dplyr
27. Auguie B. gridExtra: Miscellaneous Functions for ‘Grid’ Graphics [Internet]. 2016. Available: https://cran.r-project.org/package=gridExtra
28. Wickham H. Reshaping Data with the reshape Package. J Stat Softw. 2007;21: 1–20. Available: http://www.jstatsoft.org/v21/i12/
29. Persson, H. E., Sjöberg, G. K., Haines, J. A., de Garbino, J. P. Poisoning severity score. Grading of acute poisoning. *Journal of Toxicology: Clinical Toxicology*, *36*(3), 205-213.
30. Richards JR. Rhabdomyolysis and drugs of abuse. J Emerg Med. 2000;19: 51–6. Available: http://www.ncbi.nlm.nih.gov/pubmed/10863119
31. Goldstein RA, DesLauriers C, Burda AM. Cocaine: History, Social Implications, and Toxicity—A Review. Disease-a-Month. 2009;55: 6–38. doi:10.1016/j.disamonth.2008.10.002
32. Borek HA, Holstege CP. Hyperthermia and multiorgan failure after abuse of ‘bath salts’ containing 3,4-methylenedioxypyrovalerone. Ann Emerg Med. 2012;60: 103–5. doi:10.1016/j.annemergmed.2012.01.005
33. Liechti ME, Kupferschmidt H. Gamma-hydroxy-butyrate (GHB) and gamma-butyrolactone (GBL): analysis of overdose cases reported to the Swiss Toxicological Information Centre. Swiss Med Wkly 2004;134:534–7.

34. Liechti ME, Kupferschmidt H. Reply: Rhabdomyolysis and drugs of abuse. Swiss Med Wkly 2005, 135:206-7.
35. Liechti, M. E., Kunz, I., & Kupferschmidt, H. (2005). Acute medical problems due to Ecstasy use. Case-series of emergency department visits. Swiss medical weekly, 135(43-44), 652-657.
36. Delaney KA, Givens ML, Vohra RB. Use of RIFLE criteria to predict the severity and prognosis of acute kidney injury in emergency department patients with rhabdomyolysis. J Emerg Med. 2012;42: 521–8. doi:10.1016/j.jemermed.2011.03.008
37. Richards JR. Rhabdomyolysis and drugs of abuse. J Emerg Med. 2000;19: 51–6. Available: http://www.ncbi.nlm.nih.gov/pubmed/10863119
38. Fernandez WG, Hung O, Bruno GR, Galea S, Chiang WK. Factors predictive of acute renal failure and need for hemodialysis among ED patients with rhabdomyolysis. Am J Emerg Med. 2005;23: 1–7.
39. Grunau BE, Pourvali R, Wiens MO, Levin A, Li J, Grafstein E, et al. Characteristics and thirty-day outcomes of emergency department patients with elevated creatine kinase. O'Neil BJ, editor. Acad Emerg Med. 2014;21: 631–6. doi:10.1111/acem.12385
40. Veenstra J, Smit WM, Krediet RT, Arisz L. Relationship between elevated creatine phosphokinase and the clinical spectrum of rhabdomyolysis. Nephrol Dial Transplant. 1994;9: 637–41. Available: http://www.ncbi.nlm.nih.gov/pubmed/7970089
41. Janković SR, Stosić JJ, Vucinić S, Vukcević NP, Ercegović GV. Causes of rhabdomyolysis in acute poisonings. Vojnosanit Pregl. 2013;70: 1039–45. Available: http://www.ncbi.nlm.nih.gov/pubmed/24397200
42. Gabow PA, Kaehny WD, Kelleher SP. The spectrum of rhabdomyolysis. Medicine (Baltimore). 1982;61: 141–52. Available: http://www.ncbi.nlm.nih.gov/pubmed/7078398
43. Ward MM. Factors predictive of acute renal failure in rhabdomyolysis. Arch Intern Med. 1988;148: 1553–7. Available: http://www.ncbi.nlm.nih.gov/pubmed/3382301
44. Woodrow G, Brownjohn AM, Turney JH. The clinical and biochemical features of acute renal failure due to rhabdomyolysis. Ren Fail. 1995;17: 467–74. Available: http://www.ncbi.nlm.nih.gov/pubmed/7569117
45. Brown CVR, Rhee P, Chan L, Evans K, Demetriades D, Velmahos GC. Preventing renal failure in patients with rhabdomyolysis: do bicarbonate and mannitol make a difference? J Trauma. 2004;56: 1191–6. Available: http://www.ncbi.nlm.nih.gov/pubmed/15211124
46. de Meijer AR, Fikkers BG, de Keijzer MH, van Engelen BGM, Drenth JPH. Serum creatine kinase as predictor of clinical course in rhabdomyolysis: a 5-year intensive care survey. Intensive Care Med. 2003;29: 1121–5. doi:10.1007/s00134-003-1800-5
47. Supady A, Schwab T, Busch H-J. „Liquid Ecstasy": Gamma-Butyrolacton-Entzugsdelir mit Rhabdomyolyse und dialysepflichtiger Niereninsuffizienz. DMW - Dtsch Medizinische Wochenschrift. 2009;134: 935–937. doi:10.1055/s-0029-1220252
48. Liakoni E, Yates C, Dines AM, et al. Acute recreational drug toxicity: Comparison of self-reports and results of immunoassay and additional analytical methods in a multicenter European case series. Medicine (Baltimore). 97(5):e9784. doi:10.1097/MD.0000000000009784

Figure 1

Click here to access/download;Figure;Fig1.jpeg

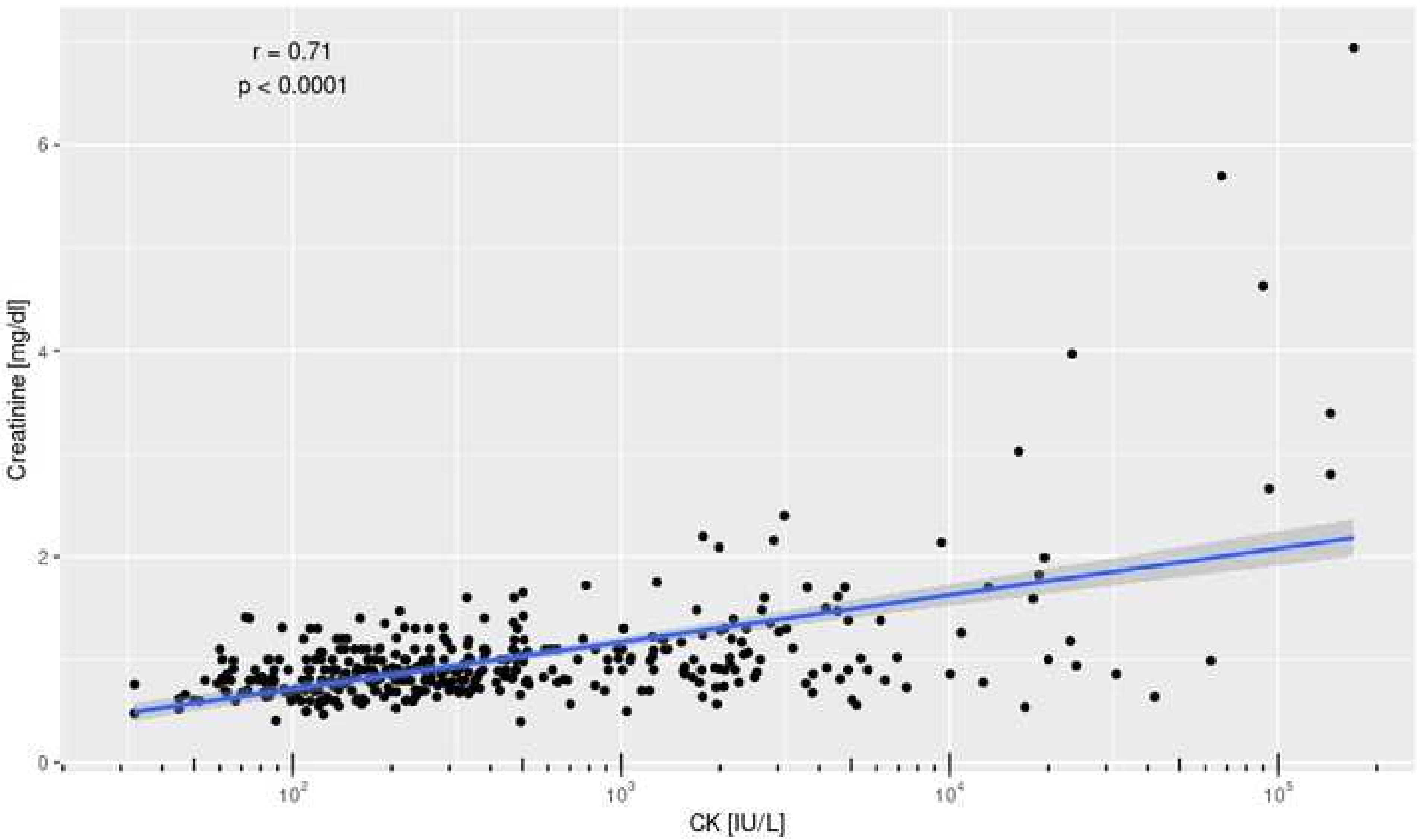

Supplement: Attachment — Submitted filename: PONE-D-20-18319_reviewer.pdf [file pone.0246297.s001.pdf]
