# Supplementary material for: Rhabdomyolysis related to acute recreational drug toxicity—A Euro-DEN study
Source: PLoS One. 2021 Mar 11;16(3):e0246297. doi: 10.1371/journal.pone.0246297 (PMC7951866; doi:10.1371/journal.pone.0246297)
Supplement: Attachment — Submitted filename: Response_to_reviewers.doc [file pone.0246297.s002.doc]

Dear Reviewers,

we would like to thank you for your review of our manuscript. We hope, that you will find our responses and revisions of the document satisfactory to reconsider the publication of our paper.

**1. Please ensure that your manuscript meets PLOS ONE's style requirements, including those for file naming.**

The style has been adjusted.

**2. We note that you have indicated that data from this study are available upon request. PLOS only allows data to be**

**available upon request if there are legal or ethical restrictions on sharing data publicly. For information on unacceptable**

**data access restrictions, please see http://journals.plos.org/plosone/s/data-availability#loc-unacceptable-data-access-**

**restrictions.**

**In your revised cover letter, please address the following prompts:**

**a) If there are ethical or legal restrictions on sharing a de-identified data set, please explain them in detail (e.g., data**

**contain potentially identifying or sensitive patient information) and who has imposed them (e.g., an ethics committee).**

**Please also provide contact information for a data access committee, ethics committee, or other institutional body to which**

**data requests may be sent.**

The dataset contains date and time of patients admission to the hospital, which could potentially identify a patient; furthermore recreational drug use is a sensitive area for many individuals and therefore the risks associated with identification are greater in this context. The data can be obtained from European Drug Emergencies Network steering committee.

The restrictions have been imposed by the EuroDEN steering committee.

For inquiries regarding data access, please contact Mrs Barbara Whetton, phone number: +44 (0)20 7188 5848 [barbara.whetton@gstt.nhs.uk](mailto:barbara.whetton@gstt.nhs.uk)

**b) If there are no restrictions, please upload the minimal anonymized data set necessary to replicate your study findings**

**as either Supporting Information files or to a stable, public repository and provide us with the relevant URLs, DOIs, or**

**accession numbers. Please see http://www.bmj.com/content/340/bmj.c181.long for guidelines on how to de-identify and**

**prepare clinical data for publication. For a list of acceptable repositories, please see**

**http://journals.plos.org/plosone/s/data-availability#loc-recommended-repositories.**

**We will update your Data Availability statement on your behalf to reflect the information you provide.**

**3. One of the noted authors is a group or consortium [Euro-DEN Research Group]. In addition to naming the author group,**

**please list the individual authors and affiliations within this group in the acknowledgments section of your manuscript.**

**Please also indicate clearly a lead author for this group along with a contact email address.**

Corrected

**4. Your ethics statement must appear in the Methods section of your manuscript. If your ethics statement is written in any**

**section besides the Methods, please move it to the Methods section and delete it from any other section. Please also**

**ensure that your ethics statement is included in your manuscript, as the ethics section of your online submission will not**

**be published alongside your manuscript.**

Corrected

**5. Please include a copy of Table 2 which you refer to in your text (line 191).**

Added

**Reviewers' comments:**

Reviewer #1: In this retrospective analysis, the authors described cases of rhabdomyolysis in patients who presnted to the Emergency Department with severe acute recreational drug toxicity. The study presents an interesting perspective on the frequency of rhabdomyolysis related to different drugs of abuse. However, the manuscript will require a major revision to make it suitable for publication. Areas that require the authors’ attention are itemized below.

*Method*

*1. Page 10 line 129-Any justification for restricting the retrospective study to Oct 2013 to Sept 2014? Are there more*

*recent data?*

Whilst Euro-DEN continued after Sept 2014 as the Euro-DEN Plus project creatinine concentration and CK activity are no longer collected therefore the data included in this study are only available for the period Oct 2013 – Sept 2014. We have clarification of this in the Materials and Methods.

*2. Page 11, line 165-168-Statistical analysis- The use of range and interquartile range as measures of data spread suggest that the data were not normally distributed. Therefore, the use of parametric test such as pearson's moment correlation and ANOVA for analysis may not be appropriated. The authors should consider the use of equivalent non parametric test for the analysis.*

Thank you – we agree, we have adjusted the statistical tests for non-normally distributed data.

*Results*

*1. Page 12, line 177-192- Correlation between CK and creatinine. This analysis is not necessary, as both biochemical parameter are the result of massive breakdown of skeletal muscles which occurs in Rhabdomyolysis. Early detection of acute renal failure can be achieved by monitoring serum creatinine and serum creatine kinase. CK is a better predictor of ARF due to rhabdomyolysis than creatinine. Since CK and creatinine are usually highly correlated in rhabsomyolysis, an attempt to find the correlation between them in this study will result in multicollinearity.*

We are aware of the high correlation between CK activity and creatinine concentration. However our clinical experience has shown, that some of patients were have very high CK activity without significant kidney injury. The serum creatinine concentration in this study was used as the marker of kidney injury. Unfortunately, due to study design (patients were treated mainly in emergency departments and only the highest recorded creatinine concentration was recorded in the dataset) we could not apply KDIGO criteria for acute kidney injury. We have addressed this in revised limitations of the paper.

*2. The result tables are not properly labelled. Also, the table do not present a clear distinction between the exposure and outcome variables making them difficult to interpret.*

Thank you - we corrected the labelling of the tables. Hopefully, this makes them more comprehensible.

*Discussion*

*1. The discussion needs to be better organized. There should be a clear pattern on how current findings connect to, support, elaborate on, or challenge conclusions of earlier studies.*

Thank you, we shortened the discussion and added connections to our study, to make it more clear and concise.

Thank you for consideration of our paper, and we hope that the revised version presents a higher standard, that would be suitable for publication.

Best regards

Piotr Maciej Kabata, on behalf of Euro-DEN Research Group
